# Visit-to-visit blood pressure variability and dementia risk after considering antihypertensive treatment: real-world data from the Japanese National Health Insurance

**DOI:** 10.1038/s41440-025-02451-1

**Published:** 2025-11-10

**Authors:** Michihiro Satoh, Hiroki Nobayashi, Shingo Nakayama, Yutaro Iwabe, Takahito Yagihashi, Seiya Izumi, Takahisa Murakami, Yuya Suzuki, Maya Toyama, Tomoko Muroya, Takayoshi Ohkubo, Hirohito Metoki

**Affiliations:** 1 Division of Public Health, Hygiene and Epidemiology, Faculty of Medicine, Tohoku Medical and Pharmaceutical University, Sendai, Japan; 2 Department of Preventive Medicine and Epidemiology, Tohoku Medical Megabank Organization, Tohoku University, Sendai, Japan; 3 Department of Pharmacy, Tohoku Medical and Pharmaceutical University Hospital, Sendai, Japan; 4 Division of Nephrology and Hypertension, Department of Internal Medicine, The Jikei University School of Medicine, Tokyo, Japan; 5 Department of Pathology and Laboratory Medicine, Cedars-Sinai Medical Center, Los Angeles, CA USA; 6 Center for Clinical Research Promotion and Development, Tohoku Medical and Pharmaceutical University Hospital, Sendai, Japan; 7 Department of Neurology, Faculty of Medicine, Tohoku Medical and Pharmaceutical University, Sendai, Japan; 8 Department of Obstetrics and Gynecology, Tohoku University Graduate School of Medicine, Sendai, Japan; 9 Division of Aging and Geriatric Dentistry, Department of Rehabilitation Dentistry, Tohoku University Graduate School of Dentistry, Sendai, Japan; 10 Division of Nephrology and Hypertension, Tohoku Medical and Pharmaceutical University, Sendai, Japan; 11 Department of Nephrology, Self-Defense Forces Sendai Hospital, Sendai, Japan; 12 Division of Internal Medicine, Izumi Hospital, Sendai, Japan; 13 Nanatsumori Family Clinic, Miyagi, Japan; 14 Department of Hygiene and Public Health, Teikyo University School of Medicine, Tokyo, Japan; 15 Tohoku Institute for the Management of Blood Pressure, Sendai, Japan

**Keywords:** Antihypertensive treatment, Blood pressure variability, Dementia, Digital hypertension, Epidemiology

## Abstract

This retrospective cohort study evaluated the association between visit-to-visit blood pressure (BP) variability and dementia risk, considering antihypertensive drug classes and medication adherence using Japanese National Health Insurance data (2015–2023) of 301,448 participants (age: 66.6 years, male: 38.6%). Visit-to-visit systolic BP (SBP) variability was assessed using the coefficient of variation (CV) from five annual health check-ups. The outcome was antidementia drug initiation as a proxy for dementia diagnosis and was analyzed using Fine-Gray models, with death as the competing outcome. For participants untreated/treated with antihypertensive medication, 366/298 initiated antidementia drugs during 2.20 ± 1.19/2.11 ± 1.19 years of follow-up, respectively. The highest SBP-CV sextile was associated with dementia risk regardless of treatment status: hazard ratio of the 6^th^ sextile (SBP-CV ≥ 9.83%) vs. 1^st^–5^th^ sextiles was 1.50 (95% confidence interval [CI]: 1.17–1.92) for untreated participants, while for treated participants, the hazard ratio of the 6^th^ sextile (SBP-CV ≥ 10.67%) was 1.43 (95%CI: 1.09–1.89) after further adjusting for antihypertensive drug classes and medication adherence assessed by medication possession rate. In the stratification analyses by baseline characteristics, only HbA1c in treated participants demonstrated a significant interaction with SBP-CV for dementia risk; this association was pronounced among treated participants with HbA1c ≥ 6.5% (interaction *P* = 0.024). No significant interactions were observed among antihypertensive drugs, poor adherence, and SBP-CV in relation to dementia risk. High visit-to-visit BP variability, indicated by a CV of approximately ≥10%, is associated with dementia, regardless of treatment status. In the treated participants, this association persisted even after accounting for antihypertensive drug classes and medication adherence.

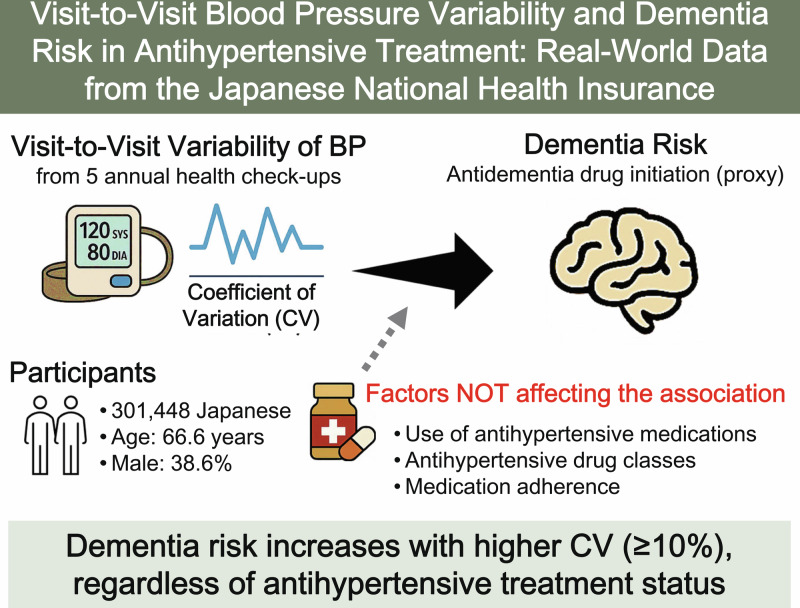

## Introduction

Dementia is a growing global health concern [1]. Identifying individuals at high risk of developing dementia to enable early prevention strategies or timely interventions is crucial. Accumulating evidence from prospective studies suggests that higher visit-to-visit blood pressure (BP) variability is associated with an increased risk of dementia and cognitive decline [2–13].

When examining the association between BP variability and dementia risk, antihypertensive treatment should be considered a potential confounding factor or effect modifier, as demonstrated in previous studies [6–12, 14]. However, few prospective studies have examined specific antihypertensive drug classes that may influence both BP variability and dementia risk [15–17]. One cohort study adjusted for drug classes when analyzing the association between visit-to-visit BP variability and dementia risk in a chronic kidney disease (CKD) population; however, medication adherence was not considered [18]. Regarding specific drug classes, only one study in the United States found a notable association among participants not taking calcium channel blockers (CCBs) [19].

Medication adherence is a crucial factor influencing the association between BP variability and dementia. However, no study has simultaneously considered medication adherence and the use of antihypertensive drug classes. Although the associations between visit-to-visit BP variability and cardiovascular disease or all-cause mortality persist after adjusting for medication adherence [20], such information regarding dementia outcomes is lacking.

The objective of this study was to evaluate the association between visit-to-visit BP variability and dementia risk. We considered antihypertensive drug classes and medication adherence when analyzing participants undergoing antihypertensive treatment.

## Methods

### Study design

This retrospective cohort study used insurance databases provided by DeSC Healthcare Inc. (Tokyo, Japan) for the period from 2014 to 2023. The DeSC database does not cover all Japanese citizens; instead, it includes claims and health check-up data (including BP) collected from multiple municipal insurers that have contracts with DeSC Healthcare Inc. A previous study has shown that the distribution of age and sex in the DeSC database was comparable to that of Japanese population estimates [21]. From the different DeSC databases, this study used only data from the National Health Insurance (NHI), as this contains information on the loss of eligibility for defining the death outcome. The NHI participants predominantly include self-employed individuals, unemployed individuals, and farmers aged <75 years [22–24]. The present study did not include any individuals aged ≥75 years old, because all insured Japanese citizens are required to switch to a Later-Stage Elderly Healthcare System upon reaching 75 years of age. Therefore, participants who reached 75 years of age during the follow-up period were censored at that point owing to loss of eligibility for inclusion in the NHI system.

The claims database includes data on all inpatient, outpatient, and pharmacy claims received by insurers. To define BP variability, we used five annual health check-ups (visits 1–5). Because most recent data are typically used for medical decision-making in clinical practice, the last checkup (visit 5) was defined as the baseline (Figure S1).

This study adhered to the Strengthening the Reporting of Observational Studies in Epidemiology guidelines [25]. The requirement for informed consent was waived because all data were fully de-identified prior to the analysis [26]. This study was approved by the Research Ethics Committee for Life Science and Medical Research of the Tohoku Medical and Pharmaceutical University (approval ID: 2024-2-015).

### Participant selection

**Fig. 1:**
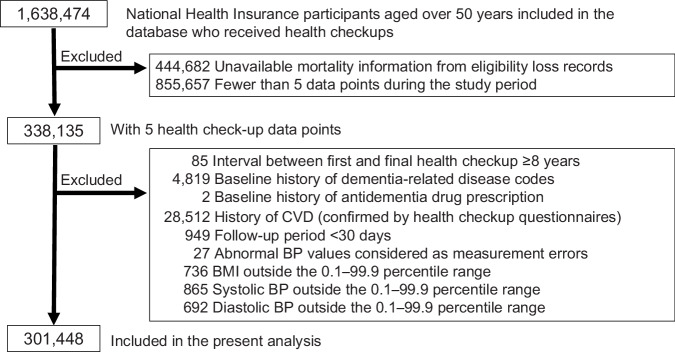
Study population selection flowchart. BMI body mass index, BP blood pressure, CVD cardiovascular disease

Figure 1 illustrates the participant-selection process. The database initially contained 1,638,474 National Health Insurance subscribers aged >50 years who underwent health checkups. We selected 338,135 patients with available mortality information from eligibility loss records and five health examination time points (not necessarily consecutive years). Of these, we excluded the following participants: 85 with intervals ≥8 years between the first and final health examinations; 4819 with dementia-related disease codes before baseline; 2 with prior antidementia drug prescriptions; 28,512 with a history of cardiovascular diseases (confirmed by health checkup questionnaires); and 949 with follow-up periods <30 days. We further excluded participants with abnormal BP considered as measurement errors, with BMI, systolic BP, or diastolic BP outside the 0.1–99.9 percentile ranges (n = 27, 736, 865, and 692, respectively). The final analytical cohort consisted of 301,448 participants.

### Drug data from claim databases

In this study, “treated” referred to participants with antihypertensive prescriptions within 365 days before the baseline, as identified from claims data. Due to the limited number of events in several drug class categories, antihypertensive medications were classified into the following five major groups (World Health Organization - Anatomical Therapeutic Chemical Classification [WHO-ATC] codes): (1) dihydropyridine calcium channel blockers (DHP-CCB) (C08CA), (2) angiotensin-converting enzyme inhibitors or angiotensin II receptor blockers (ACEI/ARB) (C09AA, C09CA), (3) thiazide/thiazide-like diuretics (C03AA, C03BA), (4) β/αβ-blockers (C07AA, C07AB, C07AG), and (5) other antihypertensive drugs, including alpha-blockers (C02CA, G04CA03), loop diuretics (C03CA01), aldosterone receptor antagonists (C03DA), potassium-sparing diuretics (C03DB), non-DHP-CCB (C08DB), renin inhibitors (C09XA), ARB-neprilysin inhibitors (C09DX04), and central-acting agents and vasodilators (C02AA, C02AB, C02AC, C02DB). Data on lipid-lowering (WHO-ATC code C10) and antidiabetic medication prescriptions (WHO-ATC code A10) were collected. For combination medications, each component was decomposed and counted separately.

Medication adherence was assessed using the medication possession rate (MPR) [27, 28]. The MPR was calculated as the cumulative days of medication supply divided by the total number of days between baseline and the first prescription within 365 days prior to baseline, as recent information is the most relevant for determining the patient characteristics. MPR values exceeding 100% were rounded to 100%. Poor adherence was defined as an MPR of <80%. The mean MPR was calculated when multiple medications were prescribed to the patients.

### BP measurements and BP variability

Japanese guidelines recommend that BP be measured twice consecutively in the sitting position during annual health checkups [29, 30], although actual measurement methods vary according to the health insurance society. In this study, the average of the two measurements was used when two measurements were available.

Visit-to-visit BP variability was defined as the coefficient of variation (CV), calculated as the ratio of the within-individual standard deviation to the within-individual mean, and expressed as a percentage (CV = [individual SD/individual mean] × 100). The annual change in BP (mmHg/year) was calculated as the slope from individual linear regression analyses of systolic BP values against examination dates, with the initial health check-up date as the reference point (time = 0).

### Outcome definitions

To define dementia outcomes based on the claims database, we used the initial prescriptions for the following antidementia drugs (WHO-ATC codes) as proxies for dementia diagnosis: donepezil (N06DA02), rivastigmine (N06DA03), galantamine (N06DA04), ipidacrine (N06DA05), and memantine (N06DX01). We did not use the initial definitive diagnosis of dementia from claims data because its reliability is unclear [22].

Death outcomes were extracted from information on the loss of eligibility as an NHI-insured person and were considered competing risks. Censoring was defined as the end of the database period or loss of eligibility for reasons other than death.

### Other baseline data

All health checkups in Japan are encouraged according to the guidelines established by the Japanese Ministry of Health, Labour, and Welfare [30]. The BMI was calculated as weight divided by height squared (kg/m²). Blood and urine samples were collected for examination. Data on HbA1c, low-density lipoprotein cholesterol (LDL), and proteinuria using the dipstick test, which are commonly used to detect metabolic syndrome in Japan, were used in this analysis. Information on smoking status, alcohol consumption, and history of cardiovascular disease was collected using questionnaires at each health checkup. Different-season checkups were defined as binary variables indicating whether any of the five health checkups were conducted in a different season from the others (summer [June–September], winter [December–March], or spring/autumn [April–May/October–November]).

### Statistical analysis

To consider the possibility of nonlinearity, we stratified the participants by sextiles of SBP-CV or DBP-CV within each untreated or treated group. Differences between groups were compared using standardized mean differences (SMDs) [31]. An SMD of |0.1| was set as the cutoff point to determine meaningful group differences [31].

Dementia risk was analyzed using Fine-Gray hazard models, with death as a competing risk [32]. Within each untreated or treated group, the sex with the lowest dementia risk was used as the reference. Hazard ratios and 95% confidence intervals (CIs) were calculated. The covariates included sex, age, BMI, smoking status, alcohol consumption, proteinuria, HbA1c, LDL, antidiabetic and lipid-lowering medication use, mean SBP, and annual SBP change. For the treated participants, the fully adjusted model further included antihypertensive drug classes (DHP-CCB, ACEI/ARB, thiazide/thiazide-like, β/αβ-blockers, and other antihypertensive drugs), use of ≥2 antihypertensive drug classes, and poor medication adherence. Missing continuous variables for HbA1c (n = 2131, 0.71%) and LDL (n = 152, 0.05%) levels were imputed using the regression slope of age, after sex stratification. Similarly, missing categorical variables for smoking status (n = 9, 0.003%), alcohol consumption (n = 13,111, 4.35%), and proteinuria (n = 436, 0.14%) were imputed to sex-specific means of the binary codes (0, 1).

Stratified analyses were conducted to assess the association between the highest SBP-CV sextile and dementia risk across baseline characteristics and follow-up duration (< 2.0/ ≥ 2.0 years). Among the treated participants, additional stratified analyses were conducted according to antihypertensive drug class, medication adherence (< 80%/ ≥ 80%), and number of antihypertensive drugs (< 2/ ≥ 2). Owing to the limited number of events in several subgroups, covariates in the stratified analyses were restricted to sex, age, and BMI. Interactions were tested using interaction terms calculated as the stratification factor multiplied by the highest SBP-CV sextile. Sex-, age-, and BMI-adjusted and fully adjusted *P* values for the interaction were calculated.

Statistical significance was set at *P* < 0.05, and all tests were two-sided. Analyses were conducted using SAS software (version 9.4; SAS Institute Inc., Cary, NC, USA).

## Results

### Baseline characteristics

Male participants comprised 38.6% of the study population, and the mean age was 66.6 ± 5.5 years. The treated participants were older and had a higher proportion of male participants than the untreated participants. Among the treated participants, DHP-CCB (72.6%) and ACEI/ARB (64.1%) were the most prescribed antihypertensive drugs, and 48.4% were prescribed ≥2 antihypertensive drugs. Other characteristics of the treated and untreated participants are presented in Supplementary Table 1.

The baseline characteristics of the participants stratified according to SBP-CV, sex, and antihypertensive treatment status are shown in Table 1. Among untreated participants, those in the highest SBP-CV sextile were older, had a lower proportion of male participants, lower BMI, higher SBP, and higher DBP compared to those in the lowest sextile, with significant differences indicated by |SMD | ≥ 0.1. Among the treated participants, those in the highest SBP-CV sextile group had a lower BMI and higher mean SBP, were more likely to use ACEIs/ARBs and multiple antihypertensive drugs, and had poorer medication adherence.

**Table 1:** Characteristics by sextiles of SBP-CV in untreated or treated participants

	Sextiles of SBP-CV in Untreated Participants, %				Sextiles of SBP-CV in Treated Participants, %			
Characteristics	S1 ≤4.14	S2–S5 4.14–9.83	S6 ≥9.83	SMD S1 vs S6	S1 ≤4.24	S2–S5 4.24–10.67	S6 ≥10.67	SMD S1 vs S6
N	31,442	125,767	31,442		18,799	75,199	18,799	
At baseline								
Male, %	37.3	35.4	32.1	-0.11	45.0	44.3	43.9	-0.02
Age, years	65.5	65.8	66.3	0.14	68.0	67.9	67.8	-0.04
BMI, kg/m^2^	22.3	22.2	22.0	-0.11	24.2	24.1	23.8	-0.11
Smoking, %	9.6	10.0	10.6	0.03	10.4	10.7	12.9	0.08
Alcohol consumption, %	19.6	19.3	18.4	-0.03	27.3	26.9	27.0	-0.01
LDL cholesterol, mg/dL	127.8	128.3	128.7	0.03	116.5	117.2	117.4	0.03
HDL cholesterol, mg/dL	67.4	67.9	68.3	0.05	62.5	62.7	62.7	0.02
HbA1c, %	5.7	5.7	5.7	0.00	5.9	5.9	5.9	-0.02
Urine protein, %	2.9	2.6	3.1	0.01	7.3	6.9	7.2	-0.00
Antidiabetic medication use, %	6.2	5.8	6.2	0.00	14.7	15.1	14.8	0.00
Lipid-lowering medication use, %	26.5	25.0	25.1	-0.03	50.4	49.2	45.6	-0.09
Systolic BP, mmHg	125.3	126.2	131.1	0.32	134.0	134.6	133.6	-0.03
Diastolic BP, mmHg	74.4	74.6	76.0	0.15	77.5	77.7	76.8	-0.07
DHP-CCB, %	-	-	-	-	74.1	72.4	71.9	-0.05
ACEI/ARB, %	-	-	-	-	60.8	63.9	68.3	0.16
Thiazide (-like) diuretics, %	-	-	-	-	7.0	7.6	8.7	0.06
β/αβ-blockers, %	-	-	-	-	9.9	9.9	11.0	0.04
Other AHT drug class, %	-	-	-	-	5.5	5.7	6.9	0.06
≥2 AHT drug class, %	-	-	-	-	46.8	47.9	52.1	0.11
Poor medication adherence, %	-	-	-	-	10.9	13.2	16.2	0.15
Based on values during five visits								
Average of SBP, mmHg	124.9	124.1	124.7	-0.01	134.0	134.8	135.8	0.16
SBP-CV, %	3.1	6.7	12.0	5.74	3.1	7.1	13.3	5.64
SBP trend, mmHg/year	0.2	0.9	2.6	0.75	0.0	0.0	-1.0	-0.07
Average of DBP, mmHg	74.4	73.9	73.6	-0.08	78.2	78.6	78.9	0.08
DBP-CV, %	5.9	7.4	10.2	1.26	5.9	7.8	11.5	1.54
DBP trend, mmHg/year	0.0	0.3	1.0	0.49	-0.4	-0.4	-1.0	-0.13
Different season checkups, %	56.2	58.6	63.8	0.15	56.4	58.6	63.4	0.14

*ACEI* angiotensin-converting enzyme inhibitor, *AHT* antihypertensive, *ARB* angiotensin II receptor blocker, *BMI* body mass index, *BP* blood pressure, *CV* coefficient of variation, *DBP* diastolic blood pressure, *DHP-CCB* dihydropyridine calcium channel blocker, *HDL* high-density lipoprotein, *LDL* low-density lipoprotein, *S* sextiles, *SBP* systolic blood pressure, *SMD* standardized mean difference

Some cut-off values were present in both S1 and S2–S5, or S2–S5 and S6, because of rounding

### BP-CV and dementia risk

During a mean follow-up of 2.20 ± 1.19 years for untreated participants and 2.11 ± 1.19 years for treated participants, antidementia drugs were initiated in 366 untreated and 298 treated participants, while 1254 and 1115 deaths occurred without experiencing the main outcome in untreated and treated participants, respectively. Of the total 664 dementia events, 23 (3.46%), 256 (38.55%), and 385 (57.98%) occurred among participants aged <65 years, 65–69 years, and ≥70 years, respectively.

**Fig. 2:**
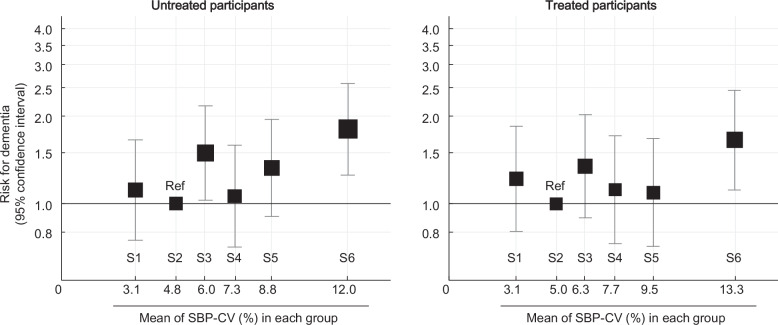
Association Between SBP-CV and Dementia Risk. The models were adjusted for age, sex, body mass index, smoking status, alcohol consumption, proteinuria, HbA1c level, low-density lipoprotein cholesterol level, antidiabetic medication use, lipid-lowering medication use, seasonal variation, average SBP, and annual SBP changes. The results for the treated participants were further adjusted for dihydropyridine calcium channel blockers, angiotensin II receptor blockers or angiotensin-converting enzyme inhibitors, thiazide/thiazide-like diuretics, β/αβ-blockers, other antihypertensive treatments, ≥2 antihypertensive drugs, and medication adherence <80%. The lowest risk group was used as the reference category. Box size indicates the number of events. SBP systolic blood pressure, S sextiles

Figure 2 shows the association between the SBP-CV sextiles and dementia risk in fully adjusted models. Regardless of the antihypertensive treatment status, the highest SBP-CV sextile consistently demonstrated the highest risk of dementia. Dementia risk across sextiles 1–5 showed no consistent patterns. Similar to the results for SBP-CV, participants in the highest sextile group of DBP-CV, with cutoff values of ≥10.79% for untreated and ≥11.57% for treated participants, had the highest risk of dementia, regardless of treatment status (Supplementary Fig. 2). The stepwise addition of covariates is presented in Supplementary Tables 2 and 3 and demonstrates the robustness of the association after sequential adjustments for potential confounders. In the fully adjusted model for the treated participants, poor medication adherence was also significantly associated with the risk of dementia (hazard ratio: 1.64, 95% CI: 1.24–2.18). The results for the analysis of the association between SBP-CV and dementia risk were similar after excluding those aged <65 years (Supplementary Table 4).

The interactions between antihypertensive medication use and the highest sextile of SBP-CV or DBP-CV on dementia risk were not significant in all participants (*P* for interaction = 0.83 and 0.55, respectively), in females, or in those aged ≥70 years or with BMI ≥ 25 kg/m² (P for interaction ≥0.20 and ≥0.47, respectively).

### Stratified analyses

Before stratification, the hazard ratio of S6 vs S1–5 was 1.54 (95%CI: 1.21–1.95) in the sex-, age-, and BMI-adjusted model and 1.50 (95%CI: 1.17–1.92) in the fully adjusted model for untreated participants, while for treated participants, it was 1.45 (95%CI: 1.10–1.90) in the sex-, age-, and BMI-adjusted model and 1.43 (95%CI: 1.09–1.89) in the fully adjusted model, including antihypertensive drug classes and medication adherence.

**Fig. 3:**
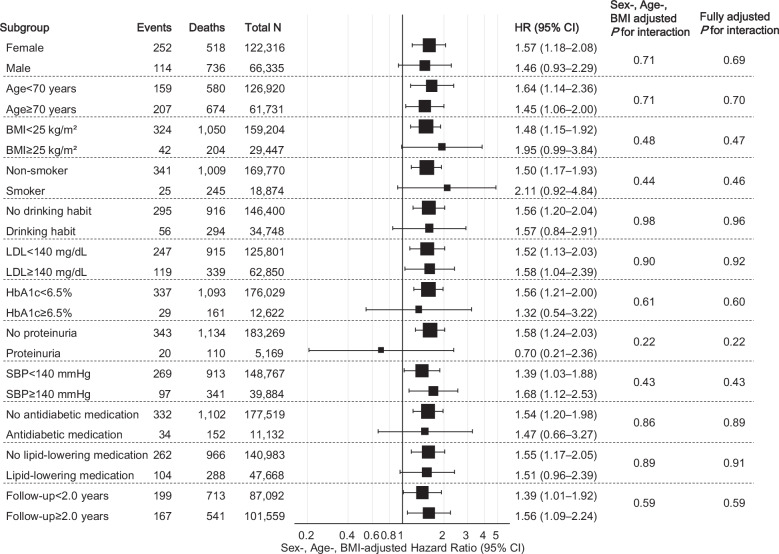
Stratified Analyses of the Association Between Highest Sextile of SBP-CV and Dementia Risk in Untreated Participants. The results indicated sex-, age-, and BMI-adjusted dementia risk for the highest sextile of SBP-CV (vs. 1^st^–5^th^ sextiles) across subgroups. Fully adjusted P-values were calculated for models with the same covariates as those shown in Fig. 2. BMI body mass index, CV coefficient of variation, SBP systolic blood pressure

**Fig. 4:**
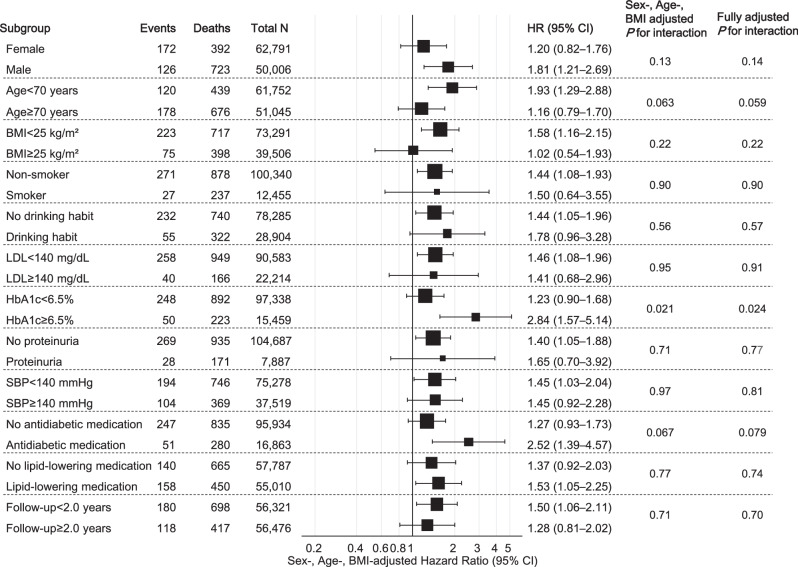
Stratified Analyses of the Association Between Highest Sextile of SBP-CV and Dementia Risk in Treated Participants. The results indicated sex-, age-, and BMI-adjusted dementia risk for the highest sextile of SBP-CV (vs. 1^st^–5^th^ sextiles) across the subgroups. Fully adjusted P-values were calculated for models with the same covariates as in Fig. 2. BMI body mass index, CV coefficient of variation, SBP systolic blood pressure

**Fig. 5:**
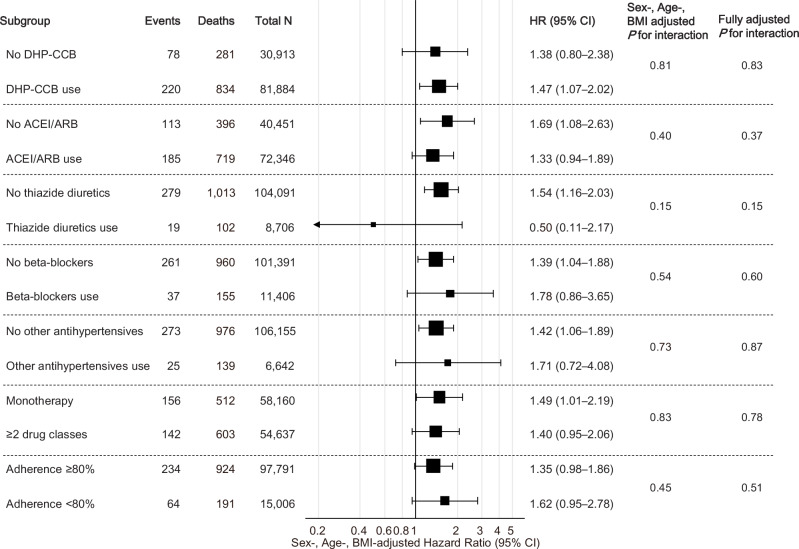
Stratified Analyses in Treated Participants According to Details of AHT. The results indicated sex-, age-, and BMI-adjusted dementia risk for the highest sextile of SBP-CV (vs. 1^st^–5^th^ sextiles) across the subgroups. Fully adjusted P-values were calculated for models with the same covariates as in Fig. 2. BMI body mass index, CV coefficient of variation, SBP systolic blood pressure, AHT antihypertensive treatment

Among the untreated participants, no significant interactions were noted between the highest sextile of SBP-CV and baseline characteristics (age, sex, BMI, smoking status, alcohol consumption, LDL, HbA1c, proteinuria, SBP, antidiabetic medication use, and lipid-lowering medication use) or a follow-up duration of 2 years on the risk of dementia (Fig. 3). Conversely, among the treated participants, significant interactions in the association between the highest sextile of SBP-CV and dementia risk were observed for sex and HbA1c levels (< 6.5%/ ≥ 6.5%), whereas the significance of the interaction by sex was diminished in the fully adjusted model (Fig. 4). Additional stratified analyses conducted by antihypertensive drug class (DHP-CCB, ACE-I/ARB, thiazide/thiazide-like diuretics, β/αβ-blockers, others), medication adherence (< 80%/ ≥ 80%), and number of antihypertensive drug classes (< 2/ ≥ 2) showed no significant interactions among the treated participants (Fig. 5).

## Discussion

In this large-scale retrospective cohort study, high visit-to-visit BP variability was significantly associated with an increased risk of dementia, regardless of the use of antihypertensive medication. Participants in the highest SBP-CV sextile, defined as ≥9.83% for the untreated group and ≥10.67% for the treated group, exhibited the greatest risk of dementia. Among participants receiving antihypertensive treatment, this association remained robust after adjusting for antihypertensive drug class and poor medication adherence. Stratified analyses revealed that this association was particularly pronounced among participants receiving antihypertensive treatment with HbA1c ≥ 6.5%. No other significant interactions were observed among the baseline characteristics, indicating a consistent relationship between BP variability and dementia.

Visit-to-visit BP variability was associated with dementia risk, although only the highest sextile, with a cutoff value of CV approximately ≥10%, revealed a significantly elevated dementia risk in both the untreated and treated groups. Our study considered antihypertensive drug classes and medication adherence, which represents the strength of this study in participants receiving antihypertensive treatment. One study demonstrated a significant association between visit-to-visit BP variability and dementia in a population with CKD after adjusting for antihypertensive drug classes, although medication adherence was not considered [18]. A post hoc analysis of a randomized controlled trial demonstrated an association between BP variability and dementia in participants receiving treatment [8]. In that study, poor BP control was identified as a potential confounding factor but was not included in the analyses. Our results indicate that medication adherence did not strongly confound the association between visit-to-visit BP variability and dementia risk in treated participants.

Our baseline characteristics suggest that DHP-CCBs may not increase visit-to-visit BP variability compared with ACEIs/ARBs, as suggested in previous studies [15–17]. However, our study did not identify any significant effect modification by the antihypertensive drug class. A study in a United States population demonstrated diminished associations between visit-to-visit BP variability and dementia among CCB users based on BP measurements taken annually over 3 or 9 years [19]. Although the cause of the differential findings between the previous study and our study remains unclear, it may be explained by the differences in age (mean: 78 vs. 66.6 years, respectively), follow-up period (approximately 20 years vs. 2 years), and ethnicity (94% Caucasian vs. Asian). Further studies are required to confirm this hypothesis. Regarding other drug classes, we observed a potential inverse association between SBP-CV and dementia risk among participants prescribed thiazide/thiazide-like diuretics. However, the limited number of participants precludes definitive conclusions, and further investigation in a larger population is required.

Among participants receiving antihypertensive treatment, the association between SBP-CV and dementia risk was particularly pronounced in those with HbA1c ≥ 6.5%. It may be crucial to pay particular attention to high visit-to-visit BP variability in patients with hyperglycemia receiving antihypertensive treatment for the early detection or prevention of dementia. Diabetes mellitus is a risk factor for neuropathy and dementia [33, 34]. Additionally, among treated individuals, vascular damage may accumulate owing to elevated BP, and atherosclerosis may subsequently develop. High visit-to-visit BP variability in patients receiving antihypertensive treatment for concurrent diabetes mellitus may strongly reflect vascular damage or neurological impairment. Meanwhile, the association between SBP-CV and dementia risk was close to null in some subgroups, such as treated participants who were female, had a BMI ≥ 25 kg/m², or were aged ≥70 years; however, no significant interactions were observed. The utility of visit-to-visit BP variability for dementia prediction may be limited in these subgroups and should therefore be interpreted with caution.

This study had several limitations. First, our study cohort was confined to individuals who completed at least five health examinations within 8 years. This potentially introduced a healthy user bias and restricted the external validity of our conclusions to the general population. The number of dementia events among participants aged <65 years was limited. Further studies are required to clarify whether visit-to-visit BP variability is associated with early-onset dementia. Second, our outcome measures relied on antidementia drug initiation, which may have overlooked patients managed using non-pharmacological cognitive interventions. Nevertheless, given that antidementia medications are seldom prescribed to cognitively intact individuals, this definition likely maintains a high specificity and supports reliable relative risk calculations [22]. Third, the relatively short follow-up duration may have been insufficient to capture the complete trajectory of cognitive decline. The transition from normal cognition to amnestic mild cognitive impairment typically requires approximately one decade [35]. Visit-to-visit BP variability could have been affected by subclinical cognitive decline that had not yet warranted antidementia drug therapy. Meanwhile, a stratified analysis based on follow-up duration demonstrated the consistency of our findings. Fourth, our definition of antihypertensive medications encompassed prescriptions indicated for conditions such as heart failure or renal insufficiency rather than hypertension alone. However, results from various stratified analyses by baseline characteristics and antihypertensive drug categories suggested that this potential misclassification had a minimal impact on our conclusions. Finally, unmeasured confounding variables, including educational attainment and socioeconomic status, which were unavailable in our database, may have influenced the observed associations.

In conclusion, high visit-to-visit BP variability, defined as CV ≥ 10%, provides significant prognostic information for dementia risk. Among participants receiving antihypertensive treatment, the results were robust after adjustment and stratification by antihypertensive drug class and adherence to medication. The association between SBP-CV and dementia risk was particularly pronounced in participants who were receiving antihypertensive treatment and had elevated HbA1c levels. Given these findings and those of previous studies, monitoring visit-to-visit BP variability may be beneficial for assessing the risk of developing dementia.

## Supplementary information

Supplemental Material

## Data Availability

The data in this study are not authorized for use by third parties under a contract with DeSC Healthcare Inc. The authors provide additional analyses upon request. The DeSC database is available to anyone who purchases it from DeSC Healthcare Inc.

## References

[1] G. B. D. Dementia Forecasting Collaborators. Estimation of the global prevalence of dementia in 2019 and forecasted prevalence in 2050: an analysis for the Global Burden of Disease Study 2019. Lancet Public Health. 2022;7:e105–25. 34998485 10.1016/S2468-2667(21)00249-8 PMC8810394

[2] Ma Y, Tully PJ, Hofman A, Tzourio C. Blood pressure variability and dementia: a state-of-the-art review. Am J Hypertens. 2020;33:1059–66. 32710605 10.1093/ajh/hpaa119

[3] Nagai M, Hoshide S, Kario K. Hypertension and dementia. Am J Hypertens. 2010;23:116–24. 19927134 10.1038/ajh.2009.212

[4] Kulkarni S, Parati G, Bangalore S, Bilo G, Kim BJ, Kario K, et al. Blood pressure variability: a review. J Hypertens. 2025;43:929–38. 40084481 10.1097/HJH.0000000000003994 PMC12052075

[5] Lee SH, Han K, Cho H, Park YM, Kwon HS, Kang G, et al. Variability in metabolic parameters and risk of dementia: a nationwide population-based study. Alzheimers Res Ther. 2018;10:110. 30368247 10.1186/s13195-018-0442-3 PMC6204276

[6] Ma, Wolters Y, Chibnik FJ, Licher LB, Ikram MA S, Hofman A, et al. Variation in blood pressure and long-term risk of dementia: A population-based cohort study. PLoS Med. 2019;16:e1002933. 31714941 10.1371/journal.pmed.1002933 PMC6850672

[7] Alperovitch A, Blachier M, Soumare A, Ritchie K, Dartigues JF, Richard-Harston S, et al. Blood pressure variability and risk of dementia in an elderly cohort, the Three-City Study. Alzheimers Dement. 2014;10:S330–7. 23954028 10.1016/j.jalz.2013.05.1777

[8] Ernst ME, Ryan J, Chowdhury EK, Margolis KL, Beilin LJ, Reid CM, et al. Long-term blood pressure variability and risk of cognitive decline and dementia among older adults. J Am Heart Assoc. 2021;10:e019613. 34176293 10.1161/JAHA.120.019613 PMC8403315

[9] Yano Y, Griswold M, Wang W, Greenland P, Lloyd-Jones DM, Heiss G, et al. Long-term blood pressure level and variability from midlife to later life and subsequent cognitive change: The ARIC neurocognitive study. J Am Heart Assoc. 2018;7:e009578. 30371241 10.1161/JAHA.118.009578 PMC6201456

[10] Yoo JE, Shin DW, Han K, Kim D, Lee SP, Jeong SM, et al. Blood pressure variability and the risk of dementia: a nationwide cohort study. Hypertension. 2020;75:982–90. 32148122 10.1161/HYPERTENSIONAHA.119.14033

[11] Qin B, Viera AJ, Muntner P, Plassman BL, Edwards LJ, Adair LS, et al. Visit-to-visit variability in blood pressure is related to late-life cognitive decline. Hypertension. 2016;68:106–13. 27217401 10.1161/HYPERTENSIONAHA.116.07494 PMC4900904

[12] den Brok M, van Dalen JW, Marcum ZA, Busschers WB, van Middelaar T, Hilkens N, et al. Year-by-year blood pressure variability from midlife to death and lifetime dementia risk. JAMA Netw Open. 2023;6:e2340249. 37902753 10.1001/jamanetworkopen.2023.40249 PMC10616718

[13] de Heus RAA, Tzourio C, Lee EJL, Opozda M, Vincent AD, Anstey KJ, et al. Association between blood pressure variability with dementia and cognitive impairment: a systematic review and meta-analysis. Hypertension. 2021;78:1478–89. 34538105 10.1161/HYPERTENSIONAHA.121.17797 PMC8516811

[14] Satoh M, Metoki H, Kikuya M, Murakami T, Tatsumi Y, Tsubota-Utsugi M, et al. Proposal of reference value for day-to-day blood pressure variability based on two outcomes: the Ohasama study. J Hypertens. 2024;42:1769–76. 38973595 10.1097/HJH.0000000000003800

[15] Wang JG, Yan P, Jeffers BW. Effects of amlodipine and other classes of antihypertensive drugs on long-term blood pressure variability: evidence from randomized controlled trials. J Am Soc Hypertens. 2014;8:340–9. 24685006 10.1016/j.jash.2014.02.004

[16] Lee JW, Choi E, Son JW, Youn YJ, Ahn SG, Ahn MS, et al. Comparison of blood pressure variability between losartan and amlodipine in essential hypertension (COMPAS-BPV). Am J Hypertens. 2020;33:748–55. 32267481 10.1093/ajh/hpaa060

[17] de Havenon A, Petersen N, Wolcott Z, Goldstein E, Delic A, Sheibani N, et al. Effect of dihydropyridine calcium channel blockers on blood pressure variability in the SPRINT trial: a treatment effects approach. J Hypertens. 2022;40:462–9. 34694261 10.1097/HJH.0000000000003033 PMC11284837

[18] Park S, Cho S, Lee S, Kim Y, Park S, Huh H, et al. Association between visit-to-visit blood pressure variability and risks of dementia in CKD patients: a nationwide observational cohort study. Clin Kidney J. 2022;15:1506–13. 36824064 10.1093/ckj/sfac020 PMC9942440

[19] Mahinrad S, Bennett DA, Sorond FA, Gorelick PB. Blood pressure variability, dementia, and role of antihypertensive medications in older adults. Alzheimers Dement. 2023;19:2966–74. 36656086 10.1002/alz.12935 PMC10354219

[20] Kronish IM, Lynch AI, Oparil S, Whittle J, Davis BR, Simpson LM, et al. The Association Between Antihypertensive Medication Nonadherence and Visit-to-Visit Variability of Blood Pressure: Findings From the Antihypertensive and Lipid-Lowering Treatment to Prevent Heart Attack Trial. Hypertension. 2016;68:39–45. 27217410 10.1161/HYPERTENSIONAHA.115.06960 PMC4900942

[21] Okada A, Yasunaga H. Prevalence of noncommunicable diseases in japan using a newly developed administrative claims database covering young, middle-aged, and elderly people. JMA J. 2022;5:190–8. 35611228 10.31662/jmaj.2021-0189 PMC9090547

[22] Satoh M, Nakayama S, Toyama M, Hashimoto H, Murakami T, Metoki H. Usefulness and caveats of real-world data for research on hypertension and its association with cardiovascular or renal disease in Japan. Hypertens Res. 2024;47:3099–113. 39261703 10.1038/s41440-024-01875-5 PMC11534704

[23] Satoh M, Murakami T, Obara T, Metoki H. Time-series analysis of blood pressure changes after the guideline update in 2019 and the coronavirus disease pandemic in 2020 using Japanese longitudinal data. Hypertens Res. 2022;45:1408–17. 35718828 10.1038/s41440-022-00961-w PMC9206892

[24] The Ministry of Health Labour and Welfare. Overview of Medical Service Regime in Japan. https://www.mhlw.go.jp/bunya/iryouhoken/iryouhoken01/dl/01_eng.pdf. Accessed 07/Aug/ 2025.

[25] von Elm E, Altman DG, Egger M, Pocock SJ, Gotzsche PC, Vandenbroucke JP, et al. The Strengthening the Reporting of Observational Studies in Epidemiology (STROBE) statement: guidelines for reporting observational studies. Lancet. 2007;370:1453–7. 18064739 10.1016/S0140-6736(07)61602-X

[26] Eba J, Nakamura K. Overview of the ethical guidelines for medical and biological research involving human subjects in Japan. Jpn J Clin Oncol. 2022;52:539–44. 35349681 10.1093/jjco/hyac034 PMC9157286

[27] Chowdhury R, Khan H, Heydon E, Shroufi A, Fahimi S, Moore C, et al. Adherence to cardiovascular therapy: a meta-analysis of prevalence and clinical consequences. Eur Heart J. 2013;34:2940–8. 23907142 10.1093/eurheartj/eht295

[28] Satoh M, Muroya T, Murakami T, Obara T, Asayama K, Ohkubo T, et al. The impact of clinical inertia on uncontrolled blood pressure in treated hypertension: real-world, longitudinal data from Japan. Hypertens Res. 2024;47:598–607. 37872377 10.1038/s41440-023-01452-2

[29] Umemura S, Arima H, Arima S, Asayama K, Dohi Y, Hirooka Y, et al. The Japanese Society of Hypertension Guidelines for the Management of Hypertension (JSH 2019). Hypertens Res. 2019;42:1235–481. 31375757 10.1038/s41440-019-0284-9

[30] The Ministry of Health Labour and Welfare. Standard health checkup and health guidance programs (2018). https://www.mhlw.go.jp/stf/seisakunitsuite/bunya/0000194155.html. Accessed 07/Aug/ 2025.

[31] Austin PC. Balance diagnostics for comparing the distribution of baseline covariates between treatment groups in propensity-score matched samples. Stat Med. 2009;28:3083–107. 19757444 10.1002/sim.3697 PMC3472075

[32] Fine JP, Gray RJ. A Proportional Hazards Model for the Subdistribution of a Competing Risk. Journal Am Stat Assoc. 1999;94:496–509.

[33] G. B. D. Nervous System Disorders Collaborators. Global, regional, and national burden of disorders affecting the nervous system, 1990-2021: a systematic analysis for the Global Burden of Disease Study 2021. Lancet Neurol. 2024;23:344–81. 38493795 10.1016/S1474-4422(24)00038-3 PMC10949203

[34] Livingston G, Huntley J, Liu KY, Costafreda SG, Selbaek G, Alladi S, et al. Dementia prevention, intervention, and care: 2024 report of the Lancet standing Commission. Lancet. 2024;404:572–628. 39096926 10.1016/S0140-6736(24)01296-0

[35] Kim KW, Woo SY, Kim S, Jang H, Kim Y, Cho SH, et al. Disease progression modeling of Alzheimer’s disease according to education level. Sci Rep. 2020;10:16808. 33033321 10.1038/s41598-020-73911-6 PMC7544693

