# Supplementary material for: Visit-to-visit blood pressure variability and dementia risk after considering antihypertensive treatment: real-world data from the Japanese National Health Insurance
Source: Hypertens Res. 2025 Nov 10;49(2):372–83. doi: 10.1038/s41440-025-02451-1 (PMC12823422; doi:10.1038/s41440-025-02451-1)
Supplement: Supplementary file 1 — Supplemental Material [file 41440_2025_2451_MOESM1_ESM.pdf]

Supplementary Materials

The authors have provided this online data supplement to readers with additional information regarding this study.

Supplement to:

Visit-to-Visit Blood Pressure Variability and Dementia Risk after Considering Antihypertensive Treatment: Real-World Data from the Japanese National Health Insurance

Correspondence :
Michihiro Satoh, PhD,
Assistant Professor (Lecturer), Division of Public Health, Hygiene and Epidemiology, Faculty of Medicine, Tohoku Medical and Pharmaceutical University, 1-15-1 Fukumuro, Miyagino-ku, Sendai, Miyagi 983-8536, Japan
E-mail: satoh.mchr@tohoku-mpu.ac.jp
Official Twitter account: @TohokuMPU_PHHE
TEL: +81-22-290-8727; FAX: +81-22-290-8728

Supplementary Table 1. Characteristics by antihypertensive treatment status

Characteristics	Total	Antihypertensive treatment		SMD Absent vs Present
		Absent	Present	
N	301,448	188,651	112,797	
At baseline				
Male, %	38.6	35.2	44.3	0.19
Age, years	66.6±5.5	65.8±5.9	67.9±4.5	0.40
BMI, kg/m^2	22.9±3.4	22.2±3.1	24.1±3.5	0.57
Smoking, %	10.4	10.0	11.0	0.03
Alcohol consumption, %	22.1	19.2	27.0	0.19
LDL cholesterol, mg/dL	124.1±31.3	128.3±30.2	117.1±31.8	-0.36
HDL cholesterol, mg/dL	65.9±17.5	67.9±17.7	62.7±16.6	-0.30
HbA1c, %	5.8±0.6	5.7±0.5	5.9±0.7	0.28
Urine protein, %	4.3	2.7	7.0	0.20
Antidiabetic medication use, %	9.3	5.9	14.9	0.30
Lipid-lowering medication use, %	34.1	25.3	48.8	0.50
Systolic BP, mmHg	129.7±16.9	126.9±17.2	134.3±15.3	0.46
Diastolic BP, mmHg	75.8±10.6	74.8±10.8	77.5±10.1	0.26
DHP-CCB, %	-	-	72.6	-
ACEI/ARB, %	-	-	64.1	-
Thiazide (-like) diuretics, %	-	-	7.7	-
β/αβ-blockers, %	-	-	10.1	-
Other AHT drug class, %	-	-	5.9	-
≥2 AHT drug class, %	-	-	48.4	-
Poor medication adherence, %	-	-	13.3	-
Based on values during 5 visits				
Average of SBP, mmHg	128.3±14.2	124.3±14.0	134.8±11.9	0.81
SBP-CV, %	7.2±3.2	7.0±3.0	7.5±3.4	0.14
SBP trend, mmHg/year	0.6±3.6	1.1±3.2	-0.2±3.9	-0.34
Average of DBP, mmHg	75.7±9.0	73.9±9.0	78.6±8.2	0.54
DBP-CV, %	7.8±3.6	7.6±3.5	8.1±3.8	0.13
DBP trend, mmHg/year	0.0±2.3	0.3±2.1	-0.5±2.4	-0.35
Different season checkups, %	59.1	59.1	59.0	0.00

ACEI, angiotensin-converting enzyme inhibitor; AHT, antihypertensive treatment; ARB, angiotensin II receptor blocker; BMI, body mass index; BP, blood pressure; CV, coefficient of variation; DBP, diastolic blood pressure; DHP-CCB, dihydropyridine calcium channel blocker; HDL, high-density lipoprotein; LDL, low-density lipoprotein; SBP, systolic blood pressure; SMD, standardized mean difference

Supplementary Table 2. Events number and stepwise adjustments for the association between SBP-CV and dementia risk

	Sextiles of SBP-CV (%)					
	S1	S2	S3	S4	S5	S6
**Untreated Participants**						
SBP-CV range, %	≤4.14	4.14–5.44	5.44–6.64	6.64–7.96	7.97–9.83	≥9.83
Events, n	52	46	69	49	62	88
Death, n	209	185	210	199	202	249
N	31,442	31,442	31,441	31,440	31,444	31,442
Model 1: Sex- and age-adjusted	1.12 (0.75–1.66)	1.00 (Ref)	1.51 (1.04–2.19)	1.07 (0.71–1.59)	1.35 (0.92–1.97)	1.90 (1.33–2.71)
Model 2: Model 1 + baseline covariates*	1.12 (0.75–1.66)	1.00 (Ref)	1.49 (1.03–2.17)	1.06 (0.71–1.59)	1.33 (0.91–1.94)	1.84 (1.29–2.62)
Model 3: Model 2 + Different season checkups + Mean SBP + SBP change	1.11 (0.75–1.66)	1.00 (Ref)	1.49 (1.03–2.17)	1.06 (0.71–1.59)	1.33 (0.90–1.95)	1.81 (1.26–2.59)
**Treated Participants**						
SBP-CV range, %	≤4.24	4.24–5.67	5.67–6.99	6.99–8.50	8.50–10.67	≥10.67
Events, n	50	40	54	44	44	66
Death, n	159	166	209	166	197	218
N	18,799	18,800	18,800	18,799	18,800	18,799
Model 1: Sex- and age-adjusted	1.21 (0.80–1.83)	1.00 (Ref)	1.36 (0.90–2.05)	1.13 (0.74–1.73)	1.13 (0.74–1.73)	1.74 (1.18–2.58)
Model 2: Model 1 + baseline covariates*	1.21 (0.80–1.84)	1.00 (Ref)	1.35 (0.90–2.03)	1.12 (0.73–1.72)	1.11 (0.73–1.71)	1.69 (1.14–2.50)
Model 3: Model 2 + Different season checkups + Mean SBP + SBP change	1.21 (0.80–1.84)	1.00 (Ref)	1.35 (0.90–2.03)	1.12 (0.73–1.72)	1.12 (0.73–1.71)	1.71 (1.15–2.54)
Model 4: Model 3 + AHT drug classes† + ≥2 AHT drugs	1.21 (0.80–1.84)	1.00 (Ref)	1.36 (0.90–2.04)	1.13 (0.74–1.73)	1.11 (0.73–1.71)	1.70 (1.14–2.53)
Model 5: Model 4 + Medication adherence<80%	1.22 (0.80–1.85)	1.00 (Ref)	1.35 (0.89–2.02)	1.12 (0.73–1.71)	1.09 (0.71–1.68)	1.66 (1.11–2.46)

Some cutoff values were present in both adjacent groups because of rounding. The results for Model 5 correspond to the values shown in Figure 2.

*Baseline covariates indicate body mass index, smoking status, alcohol consumption, proteinuria, HbA1c, low-density lipoprotein cholesterol, antidiabetic medication use, lipid-lowering medication use. †AHT drug classes include dihydropyridine calcium channel blockers, angiotensin II receptor blockers/angiotensin-converting enzyme inhibitors, thiazide/thiazide-like diuretics, β/αβ-blockers, and other AHT.

AHT, antihypertensive treatment; CI, confidence interval; SBP, systolic blood pressure; CV, coefficient of variation.

Supplementary Table 3. Events number and stepwise adjustments for the association between DBP-CV and dementia risk

	Sextiles of DBP-CV (%)					
	S1	S2	S3	S4	S5	S6
**Untreated Participants**						
DBP-CV range, %	≤4.38	4.38–5.79	5.79–7.09	7.09–8.58	8.58–10.79	≥10.79
Events, n	44	59	68	48	67	80
Death, n	181	194	217	201	212	249
N	31,437	31,441	31,447	31,444	31,439	31,443
Model 1: Sex- and age-adjusted	1.00 (Ref)	1.32 (0.90–1.96)	1.52 (1.04–2.22)	1.10 (0.73–1.65)	1.51 (1.03–2.21)	1.86 (1.28–2.68)
Model 2: Model 1 + baseline covariates*	1.00 (Ref)	1.32 (0.89–1.95)	1.51 (1.03–2.20)	1.09 (0.72–1.64)	1.48 (1.01–2.16)	1.81 (1.25–2.61)
Model 3: Model 2 + Different season checkups + Mean DBP + DBP change	1.00 (Ref)	1.32 (0.90–1.96)	1.53 (1.05–2.24)	1.12 (0.75–1.70)	1.54 (1.05–2.25)	1.90 (1.32–2.76)
**Treated Participants**						
DBP-CV range, %	≤4.58	4.58–6.10	6.10–7.49	7.49–9.14	9.14–11.57	≥11.57
Events, n	50	39	42	47	51	69
Death, n	157	153	167	194	193	251
N	18,799	18,798	18,801	18,801	18,801	18,797
Model 1: Sex- and age-adjusted	1.26 (0.83–1.92)	1.00 (Ref)	1.09 (0.70–1.68)	1.22 (0.80–1.86)	1.33 (0.88–2.01)	1.83 (1.24–2.71)
Model 2: Model 1 + baseline covariates*	1.27 (0.84–1.93)	1.00 (Ref)	1.08 (0.70–1.67)	1.21 (0.79–1.85)	1.31 (0.87–1.99)	1.81 (1.22–2.68)
Model 3: Model 2 + Different season checkups + Mean DBP + DBP change	1.26 (0.83–1.92)	1.00 (Ref)	1.09 (0.71–1.69)	1.23 (0.80–1.88)	1.35 (0.89–2.05)	1.91 (1.28–2.86)
Model 4: Model 3 + AHT drug classes† + ≥2 AHT drugs	1.27 (0.83–1.93)	1.00 (Ref)	1.10 (0.71–1.70)	1.23 (0.80–1.88)	1.35 (0.89–2.05)	1.90 (1.27–2.85)
Model 5: Model 4 + Medication adherence<80%	1.27 (0.84–1.94)	1.00 (Ref)	1.09 (0.71–1.69)	1.21 (0.79–1.86)	1.34 (0.88–2.04)	1.86 (1.24–2.80)

Some cut-off values were present in both adjacent groups because of rounding. The results of Model 5 correspond to the values shown in Supplementary Figure 2.

*Baseline covariates indicate body mass index, smoking status, alcohol consumption, proteinuria, HbA1c, low-density lipoprotein cholesterol, antidiabetic medication use, lipid-lowering medication use. †AHT drug classes include dihydropyridine calcium channel blockers, angiotensin II receptor blockers/angiotensin-converting enzyme inhibitors, thiazide/thiazide-like diuretics, β/αβ-blockers, and other AHT.

AHT, antihypertensive treatment; CI, confidence interval; DBP, diastolic blood pressure; CV, coefficient of variation.

Supplementary Table 4. Association between SBP-CV and dementia risk in participants aged ≥65 years old

	Sextiles of SBP-CV (%)					
	S1	S2	S3	S4	S5	S6
**Untreated Participants**						
SBP-CV range, %	≤4.14	4.14–5.44	5.44–6.64	6.64–7.96	7.97–9.83	≥9.83
Events, n	48	42	66	48	59	88
Death, n	181	165	169	168	168	213
N	21,495	21,526	21,634	21,822	22,344	23,005
Model 1: Sex- and age-adjusted	1.13 (0.74–1.70)	1.00 (Ref)	1.58 (1.07–2.32)	1.14 (0.76–1.73)	1.40 (0.94–2.08)	2.06 (1.43–2.98)
Model 2: Model 1 + baseline covariates*	1.13 (0.74–1.71)	1.00 (Ref)	1.56 (1.06–2.30)	1.14 (0.75–1.72)	1.38 (0.93–2.05)	1.99 (1.38–2.87)
Model 3: Model 2 + Different season checkups + Mean SBP + SBP change	1.12 (0.74–1.70)	1.00 (Ref)	1.57 (1.06–2.31)	1.14 (0.75–1.73)	1.38 (0.93–2.06)	1.96 (1.35–2.85)
**Treated Participants**						
SBP-CV range, %	≤4.24	4.24–5.67	5.67–6.99	6.99–8.50	8.50–10.67	≥10.67
Events, n	49	37	53	43	42	66
Death, n	142	154	190	147	178	197
N	15,859	15,771	15,751	15,601	15,762	15,588
Model 1: Sex- and age-adjusted	1.28 (0.83–1.96)	1.00 (Ref)	1.44 (0.95–2.20)	1.20 (0.77–1.86)	1.16 (0.75–1.81)	1.88 (1.26–2.81)
Model 2: Model 1 + baseline covariates*	1.28 (0.84–1.97)	1.00 (Ref)	1.43 (0.94–2.18)	1.19 (0.77–1.85)	1.15 (0.74–1.78)	1.83 (1.22–2.73)
Model 3: Model 2 + Different season checkups + Mean SBP + SBP change	1.28 (0.84–1.96)	1.00 (Ref)	1.43 (0.94–2.18)	1.19 (0.77–1.85)	1.15 (0.74–1.79)	1.85 (1.23–2.76)
Model 4: Model 3 + AHT drug classes† + ≥2 AHT drugs	1.28 (0.84–1.97)	1.00 (Ref)	1.44 (0.95–2.19)	1.20 (0.77–1.85)	1.15 (0.74–1.79)	1.84 (1.23–2.76)
Model 5: Model 4 + Medication adherence<80%	1.29 (0.84–1.97)	1.00 (Ref)	1.43 (0.94–2.17)	1.18 (0.76–1.83)	1.12 (0.72–1.75)	1.79 (1.19–2.68)

Some cut-off values were present in both adjacent groups because of rounding. The results for Model 5 correspond to the values shown in Figure 2.

*Baseline covariates indicate body mass index, smoking status, alcohol consumption, proteinuria, HbA1c, low-density lipoprotein cholesterol, antidiabetic medication use, lipid-lowering medication use. †AHT drug classes include dihydropyridine calcium channel blockers, angiotensin II receptor blockers/angiotensin-converting enzyme inhibitors, thiazide/thiazide-like diuretics, β/αβ-blockers, and other AHT.

AHT, antihypertensive treatment; CI, confidence interval; SBP, systolic blood pressure; CV, coefficient of variation.

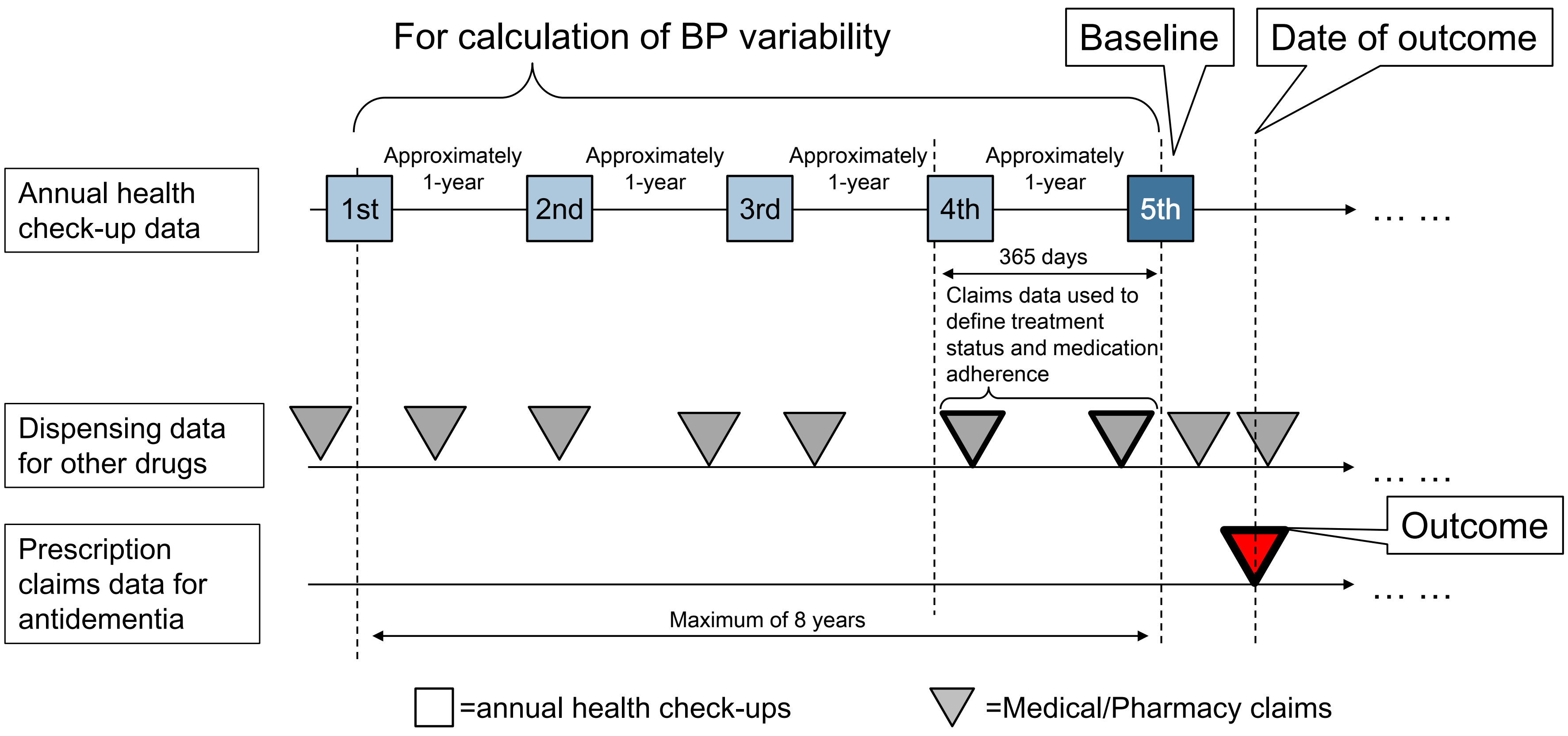

Supplementary Figure 1. Scheme of Study Design

The fifth health check-up served as the baseline, with antidementia treatment status defined using prescription claims data within 365 days following baseline. The maximum period for observing visit-to-visit blood pressure variability was 8 years.

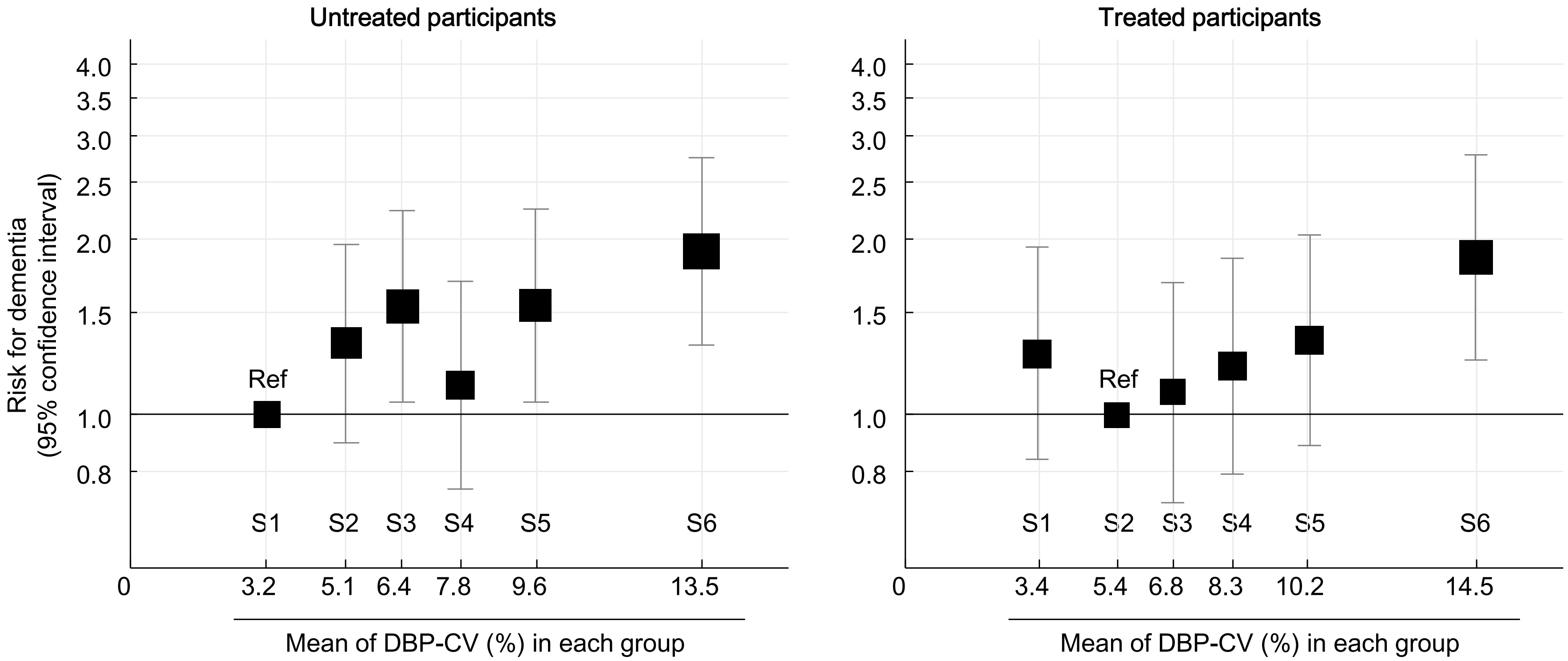

Supplementary Figure 2. Association Between DBP-CV and Dementia Risk

The models included age, sex, body mass index, smoking status, alcohol consumption habits, proteinuria, HbA1c level, low-density lipoprotein cholesterol level, antidiabetic medication use, lipid-lowering medication use, seasonal variation, average DBP, and annual DBP change as covariates. The results were further adjusted for dihydropyridine calcium channel blockers, angiotensin II receptor blockers or angiotensin-converting enzyme inhibitors, thiazide/thiazide-like diuretics, β/αβ-blockers, other antihypertensive treatments, ≥2 antihypertensive drugs, and medication adherence <80% for treated participants. The lowest risk group was used as the reference category. Box size indicates the number of events.

DBP, diastolic blood pressure; S, sextiles
